# Chemically synthesized biofuels from agricultural waste: Optimization operating parameters with surface response methodology (CCD)

**DOI:** 10.1016/j.mex.2017.09.005

**Published:** 2017-10-16

**Authors:** Tesfay Berhe, Omprakash Sahu

**Affiliations:** a Department of Chemical Engineering, KiOT, Wollo University Ethiopia, Ethiopia; b School of Chemical and Food Engineering, BiT Bahir Dar University, Ethiopia

**Keywords:** Surface response methodology for process optimization, Bioethanol, Distillation, Fermentation, Hydrolysis, Waste

## Abstract

Bioethanol is one of the most important alternative renewable energy sources that substitute the fossil fuels. Sugarcane bagasse has a content of cellulose and hemicelluloses, which make it suitable as fermentation substrate when hydrolyzed. The objective of work is ethanol production from sugarcane bagasse (SCB) by the fermentation process. Eight laboratory experiments were conducted to produce bioethanol from sugarcane bagasse. By using Design Expert, it was formulated the dilute acid hydrolysis step to investigate the effects of hydrolysis parameters on a yield of ethanol and optimum condition. All the three hydrolysis parameters were significant variables for the yield of ethanol. The optimum combinations of the three factors maximum ethanol yield were 10.86 ml at 50 g sample, 92.59 °C hydrolysis temperature, 30 min hydrolysis time and 1%v/v acid concentration. From this study following point were concluded:

• Lignocellulosic containing material are sustainable for clean energy production
• Production of bioethanol from waste sugarcane baggage’s is possible
• Operating parameters (time, temperature and acid concentration) can be optimized by surface response methodology.
• Process parameters hydrolysis, fermentation and distillation have significant role on bioethanol yield.

## Graphical abstract

**Figure:**
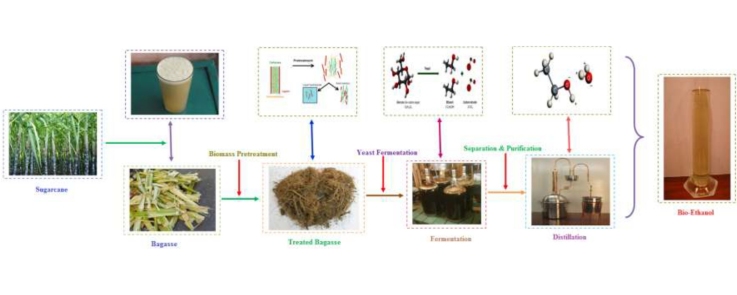

## Method overview

Energy consumption has increased steadily over the last century as the world population has grown and more countries have become industrialized. Crude oil has been the major resource to meet the increased energy demand [1], [2]. As energy demand increases the global supply of fossil fuels cause harm to human health and contributes to the greenhouse gas emission. At the same time, increasing waste generation linked to rising population and living standards is a worldwide challenge to waste management systems. World energy consumption is predicted to increase by 50% to 2030 according to the United States Energy Information Agency [3]. The combustion of fossil fuel is responsible for 73% of the CO_2_ emission [4]. In this scenario, renewable sources might serve as an alternative. The alternative fuel must be technically feasible, economically competitive, environmentally acceptable, and readily available [5]. Numerous potential alternative fuels have been proposed, including bioethanol, biodiesel, methanol, hydrogen, boron, natural gas, liquefied petroleum gas (LPG), Fischer–Tropsch fuel, p-series, electricity, and solar fuels [6]. Biofuel is those which include bioethanol, vegetable oils, biodiesel, biogas, biosynthetic gas (bio-syngas), bio-oil, biochar, Fischer–Tropsch liquids, and biohydrogen offers many advantages over petroleum-based fuels [7]. The advantages of biofuel are easily available from common biomass sources, represent a CO_2_-cycle in combustion and economical [8]. Bioethanol has the major applications in automobile, beverage, pharmaceuticals industry etc due to economically as well as ecofriendly. Bioethanol is appropriate for the mixed fuel in the gasoline engine due to its high octane number, due to its low cetane number and high heat of vaporization impede self-ignition in the diesel engine [9]. Absolute ethanol and 95% ethanol are themselves act as good solvent, somewhat less polar than water and used in perfumes, paints, and tinctures. Ethanol is used in medical wipes and in most common antibacterial hand sanitizer gels at a concentration of about 62%.

In past various material like sugarcane juice and molasses [1], [10], sugar beet, beet molasses [11], sweet sorghum [12] and starchy materials like sweet potato [13], Prosopis juliflora [14], corn cobs and hulls [15], [16], cellulosic materials like cocoa, pineapples and sugarcane waste [17], coffee husk [18] and milk/cheese/whey using lactose hydrolyzing fermenting strains has been used for bioethanol production. However, another alternative material has been used which contain lignocellulosic such as weed, grass, sawdust, municipal solid waste, woody biomass, and paper mill waste [19]. In present research work bioethanol were produced from waste sugarcane bagasse by using S. Cerevisiae as catalyst. The parameters acid concentration, working temperature and contact time has been optimized with central composite design method for the highest possible ethanol yield.

## Methodological protocols

### Material

The sugarcane bagasse was collected from sugar industry and cleans it by distilled water to remove impurity. Washed sugarcane baggage (SCB) was dried in an oven at 60 °C for 48 h [20]. The dried sample was placed in a mortar crusher to make maximum particle sizes of 3 mm. Laboratory grade sulfuric acid (H_2_SO_4_ 98%), sodium hydroxide (NaOH) solution, dry instant yeast (Saccharomyces Cerevisiae), yeast extracts (agar), urea, dextrose sugar; (MgSO_4_·7H_2_O), distilled water, and potassium dichromate were used without any purification.

### Methods

#### Media preparation

A 100 ml of media containing 10 gm Sugar (Dextrose), 0.2 gm Yeast extract, 1.0gm Urea, and 1.0 g MgSO_4_·7H_2_O was prepared [21]. The prepared media sample was sterilized in the autoclave and 0.5 gm of yeast, *Saccharomyces Cerevisiae* was added in a 250 ml conical flask and covered with aluminum foil. The conical flasks were then placed in a shaking incubator for 24 h at a temperature of 30 °C and 200 rpm [17].

#### Steam pretreatment

**Fig. 1:**
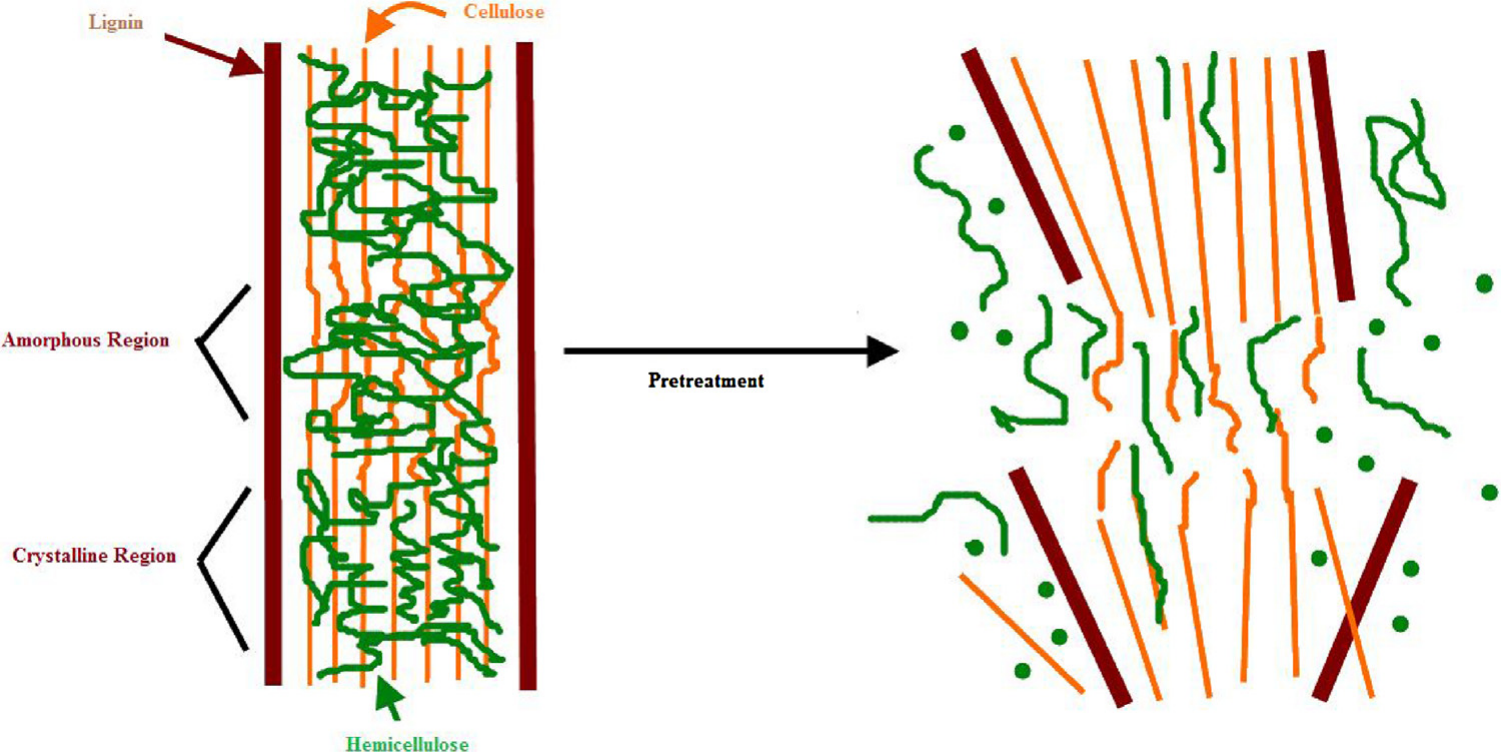
Pretraetment of sugarcane bagasse.

Pretreatment is one of significant step for maximum conversion of ethanol, Fig. 1 representing the effect of pretreatment on SCB. It especially concern to delignification of the sugarcane bagasse in order to make cellulose more accessible in the hydrolysis step Kang et al. [34]. First distilled water was prepared and then 50 g of the cut sample was soaked in a distilled water of 500 ml in conical flasks for 24 h. The conical flasks were capped with the help of aluminum foil. Then the lignocellulosic biomass was rapidly heated at 121 °C by high-pressure steam without addition of any chemicals in autoclave [22]. The biomass and steam mixture was held for 15 min to promote hydrolysis. After finishing the given pretreatment time and temperature the sample in autoclave was allowed to cool and the soluble portion was separated from the non-soluble portion. The non-soluble portion was hydrolyzed in the next steps and the soluble solution was placed in another conical flask.

#### Acid hydrolysis

In acid hydrolysis cellulose and hemicellulose of biomass were converted into glucose. The alteration of cellulose and hemicellulose can be expressed by the reaction of glucan (for hexoses) and xylan (for pentose) with water (shown in Eqs. (1) and (2)). In this step sulfuric acid (by volume to water) was diluted to 1% and 5% concentration prepared and sugarcane bagasses of 10% W/V were added into the glass vessel [23]. Then the prepared sample was hydrolyzed in autoclave with the vessels unlidded between 80 and 100 °C for 30 to 60 min. Centrifugation and then filtration was used to separate the solid particles from the liquid in the hydrolyzate (remove the non-fermentable lignin portion). The diluted hydrolyzed samples were conditioned to temperature of 30 °C before fermentation step is started. This was the temperature at which all fermentation experiments were carried out [24].

(1) (C_6_H_10_O_5_)_n_ + n(H_2_O) → nC_6_H_12_O_6_

(2) (C_5_H_8_O_4_)_n_ + n(H_2_O) → nC_5_H_10_O_5_

#### Fermentation

The supernatant from dilute acid hydrolysis of lignocelluloses can contain both six-carbon (hexoses) and five-carbon (pentoses) sugars (if both cellulose and hemicellulose are hydrolyzed). Depending on the lignocelluloses source, the hydrolysate typically consists of glucose, xylose, arabinose, galactose, mannose, fucose, and rhamnose. Microorganisms can be used to ferment all lignocellulose-derived sugars to bioethanol [25]. The conversion of hexoses and pentose into ethanol in presence of microorganism is shown in Eqs. (3) and (4).

(3) C_6_H_12_O_6_ → 2C_2_H_5_OH + 2CO_2_

(4) 3C_5_H_10_O_5_ → 3C_2_H_5_OH + 5CO_2_

#### Distillation

A distillation system was used to separates the bioethanol from water in the liquid mixture. All distillation experiments were carried out at a temperature of 85°C and a distillation time of 3 h by rotary evaporator [26].

#### Experimental method

**Fig. 2:**
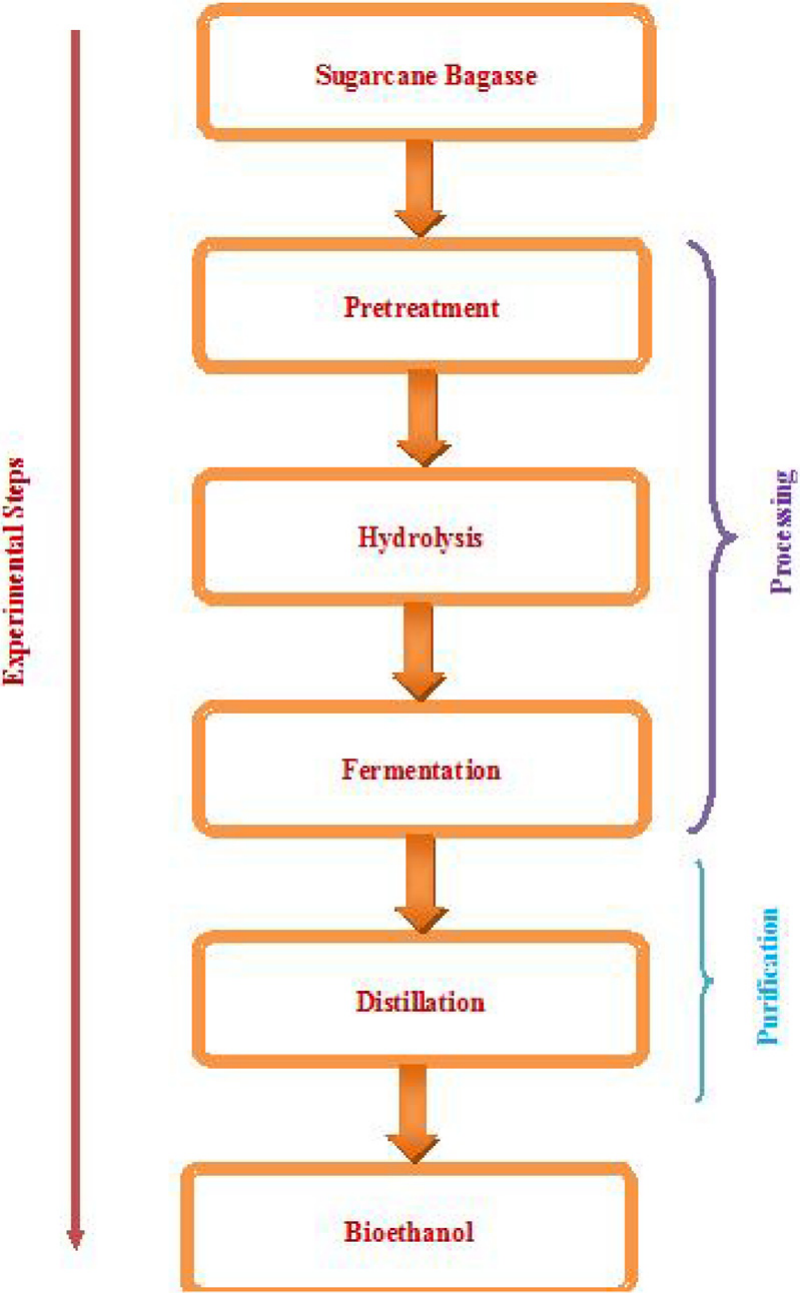
Experimental steps in bioethanol production.

A 50 g measure weight of SCB was soaked in a distilled water of 500 ml in conical flasks for 24 h. The conical flasks were capped with the help of aluminum foil. Then the lignocellulosic biomass was rapidly heated at 121 °C by high-pressure steam without the addition of any chemicals in the autoclave. The biomass/steam mixture was held for 15 min to promote hemicellulose hydrolysis. After finishing the given pretreatment time and temperature the sample in the autoclave was allowed to cool and the soluble portion was separated from the non-soluble portion. The non-soluble portion was hydrolyzed with 1% and 5% dilute sulfuric acid diluted (to maintain pH 5) in the autoclave between 80 and100 °C for 30 to 60 min. Centrifugation and then filtration was used to separate the solid particles from the liquid in the hydrolyzate (remove the non-fermentable lignin portion). Fermentation was carried out in shaking incubator. The shaking incubator was set at 30 °C and the prepared samples were dipped into the water-filled-beaker until the temperature became equal. The yeast *Saccharomyces Cerevisiae* culture was added with the proportion of 1:10 to the hydrolyzed sample. The vessel was lidded with a piece of ginned cotton covered with aluminum foil. Fermentation was let take place. After 72 h of fermentation, the sample was taken out and distilled to separate the water from ethanol. Complete bioethanol production processing is shown in Fig. 2.

#### Experimental design and data analysis

The central composite design (CCD) was employed to fit a second-order polynomial model and to obtain an experimental error. Three-parameter temperature (X_1_), time (X_2_) and acid concentration (X_3_) and two-level (2^3^ = 8) factorial design were applied to optimize the hydrolysis of ethanol yield (Y) by using design expert 7(trial version). The significance of the result was set from analysis of variance (ANOVA). The maximum and minimum values of hydrolysis parameters selected for the experiment is mention in Table 1. Modeling can be done by doing only a minimum number of experiments. In the modeling, it is not required to know the detailed reaction mechanism. The response and the corresponding parameters are modeled and optimized using analysis of variance (ANOVA). It is used to calculate the statistical parameters by means of response surface methods. Basically, this optimization process involves three major steps, which are, performing the statistically designed experiments, determining the coefficients in a mathematical model and predicting the response and checking the accuracy of the model [27]. The response can be represented as a function of variables as in Eq. (5):

(5) $$Y=f(x_{1},x_{2},x_{3}.........x_{n})$$

Where *Y* is the response of the system, and *x_i_* is the variables of action called factors. The aim is to optimize the response variable (*Y*), for this work yield of ethanol. It is assumed that the independent variables are continuous and controllable by experiments with negligible errors [28]. Total 8 experiments were carried out in this research work, for each hydrolysis parameters (temperature, time and acid concentration), at four high and four low levels in the design. The tabulated numeric representation of the factorial design is mention in Table 2.

**Table 1:** Minimum and maximum value of parameters.

S.No	Experiments	Minimum	Maximum
1	Hydrolysis temperature(°C)	80	100
2	Hydrolysis time(minutes)	30	60
3	Acid concentration(% by volume of distilled water)	1	5

**Table 2:** Numeric values of parameters in hydrolysis according to factorial design.

Run No	Temperature (X_1_)	Time (X_2_)	Concentration (X_3_)	Yield of ethanol (Y)
1	80	30	1	8.48
2	80	30	5	11.13
3	80	60	1	10.335
4	80	60	5	13.25
5	100	30	1	11.925
6	100	30	5	12.19
7	100	60	1	13.515
8	100	60	5	7.42

### Biochemical analysis

#### Identification of bioethanol

About 5 ml fermented sample was taken and the pinch of potassium dichromate and a few drops of H_2_SO_4_ were added. The color change from pink to green indicated the presence of bioethanol.

#### Determination of ethanol concentration

The ethanol concentration was determined by gas chromatography. Gas chromatograph (DANI-GC 1000) equipped with flame ionization detector (FID) was employed for the separation and quantification of ethanol. A fused silica capillary column (30 m 0.32 mm) coated with 95% methylpolysiloxane (stationary phase) was fitted into the instrument to provide on column injection. The injector and detector temperature were maintained at 210 and 250 °C, respectively. The oven starting temperature was 50 °C, one minute hold time with heating rate of 30 °C per minute to 155 °C. Nitrogen was used as carrier gas at a flow rate of 0.5 bar and for H_2_ at 0.65 bar was adjusted. The concentration of ethanol in the samples was determined using *iso*-isopropanol as internal standard [29].

## Method validation

### Fitting of second-order polynomial equation

The RSM have several classes of designs, with their own properties and characteristics. The Central composite design was used to study the effects of the variables towards their responses and subsequently in the optimization studies [30]. Experiments according to the design were carried out and relevant results are shown in Table 2. Percentage of ethanol was selected as the dependent variable. The response variable was fitted by a second-order model in the form of the quadratic polynomial equation:

(6) $$Y=b_{0}+\sum_{i=1}^{n}b_{i}x_{i}+\sum_{i=1}^{n}b_{ii}{x_{i}}^{2}\;\;+\sum_{i=1}^{n}\sum_{j>1}^{n}b_{ij}x_{i}x_{j}$$

where *Y* is the predicted response, *b*_0_ the constant coefficient, *b_i_* the linear coefficients, *b_ii_* the quadratic coefficients, *b_ij_* the interaction coefficients, and *x_i_*, *x_j_* are the coded values of the adsorption variables. The regression equation obtained in terms of coded factors for ethanol yield in percentage (Y) is presented in the below:

(7) Ethanol Yield (Y) = *11.03* *+* *0.23X_1_* *+* *0.099X_2_ − 0.033X_3_ − 0.89X_1_X_2_ − 1.42X_1_X_3_ − 0.76X_2_X_3_*

The adequacy of the generated regression model was also evaluated using ANOVA method, which is very useful to determine significant effects of process variables to the response and to fit the second order polynomial models to the experimental data [31]. Table 3 shows the outcome of such an analysis. The probability (P-values) values were used as a device to check the significance of each coefficient, which also showed the interaction strength of each parameter. The smaller the P-values are, the bigger the significance of the corresponding coefficient. F- Value is a test for comparing model variance with residual (error) variance. If the variances are close to the same, the ratio will be close to one and it is less likely that any of the factors have a significant effect on the response. It was calculated by Model Mean Square divided by Residual Mean Square. Here the Model F-value of 7.60 implies the model is significant. There is only a 0.40% chance that a “Model F-Value” this large could occur due to noise. Values of “Prob > F” less than <0.1000 indicate model terms are significant. In this case, X_1_X_2_, X_1_X_3_, X_2_X_3_ are significant model terms. Values greater than 0.1000 indicate the model terms are not significant. The coefficient of variation, the standard deviation expressed as a percentage of the mean, the values of Adj-R^2^ and Pre- R^2^ were evaluated as 0.7252 and 0.5362, respectively, showing a very good agreement between the predicted and actual data.

**Table 3:** Analysis of variance (ANOVA) for the factorial model.

Source	Sum of Squares	‘df	Mean Square	F Value	P-value Prob > F	Remark
Model	55.58	6	9.26	7.60	0.0040	Significant
X_1_	0.86	1	0.86	0.71	0.4227	
X_2_	0.16	1	0.16	0.13	0.7272	
X_3_	0.018	1	0.018	0.014	0.9071	
X_1_X_2_	12.80	1	12.80	10.50	0.0102	Significant
X_1_X_3_	32.46	1	32.46	26.63	0.0006	Significant
X_2_X_3_	9.29	1	9.29	7.62	0.0221	Significant
Residual	10.97	9	1.22			
Lack of Fit	10.97	1	10.97			
Pure Error	0.000	8	0.000			
Cor Total	66.56	15				

#### Effect of temperature and time

**Fig. 3:**
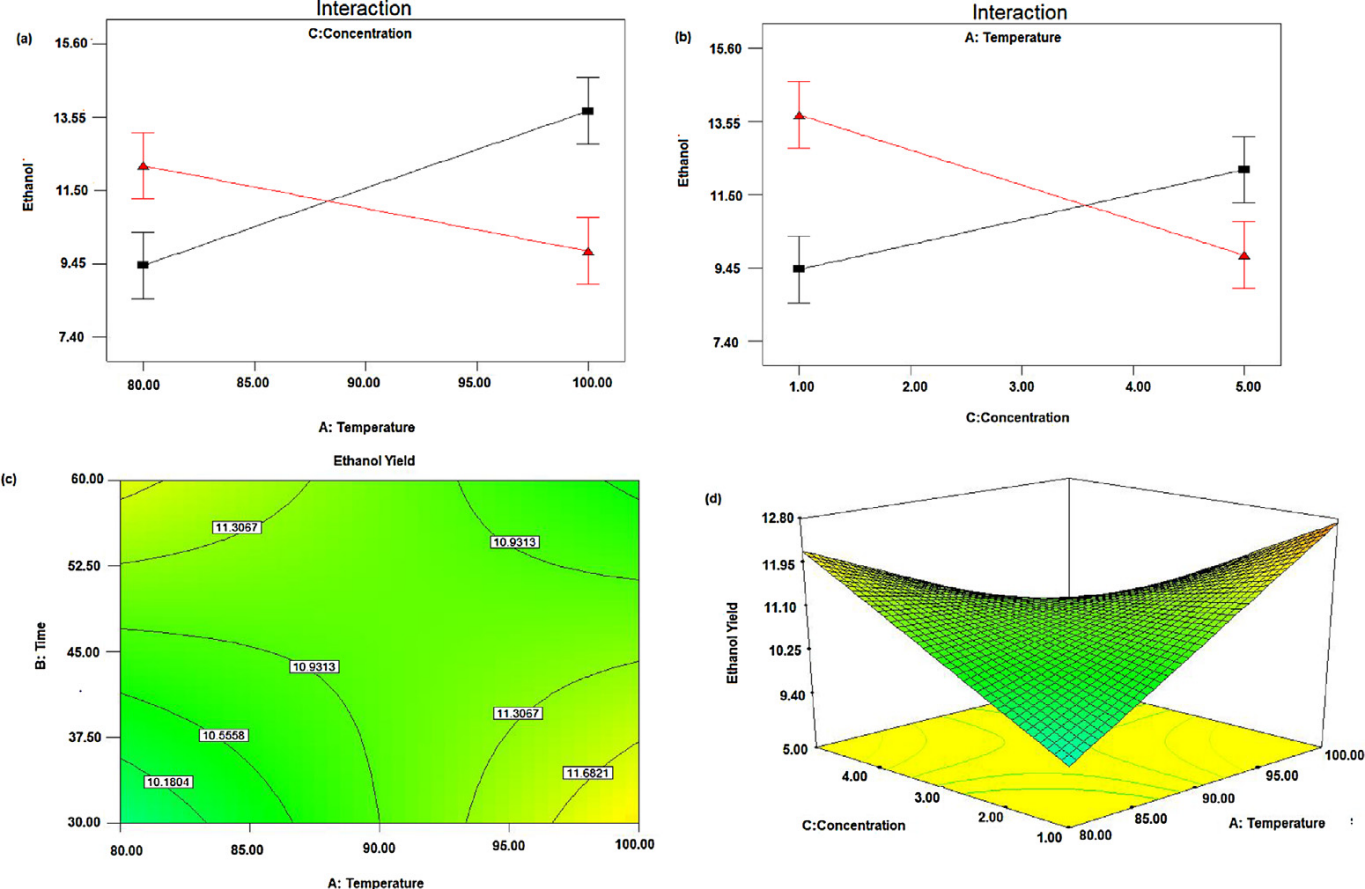
Effect of temperature and time on yield of ethanol when acid concentration was at the center point (a) effects of temperature and time (fixed), (b) effects of temperature (fixed) and time, (c) Contour plots and (d) Response surfaces plot.

The effect of temperature and time is represented in Fig. 3(a) and (b), it shows an effect of temperature and time on the yield of ethanol when the acid concentration was at the center point. Ethanol yield increased with increasing hydrolysis temperature when hydrolysis time was at a low level. Similarly, ethanol yield increased with increasing hydrolysis time when hydrolysis temperature was at a low level. This may be due to low temperature and time cellulose might not be converted to fermentable sugars and at high temperature and time, the fermentable sugars might be converted to non-fermentable molecules [32]. Hence both temperature and time have interaction effect, in addition to the main effect for the yield of ethanol production. The contour plot graph showing predicted the response of ethanol yield as a function of hydrolysis time and hydrolysis temperature, which is shown in Fig. 3(c). Ethanol yield increased as hydrolysis time increases at the lower level temperature and it decrease when the hydrolysis time and temperature became higher and higher. The response surface Fig. 3(d), obtained from hydrolysis temperature and time shows that ethanol yield increased with increasing the time when hydrolysis temperature was at low level and with increasing hydrolysis temperature when the time was at the low level [33].

#### Effect of acid concentration and time

**Fig. 4:**
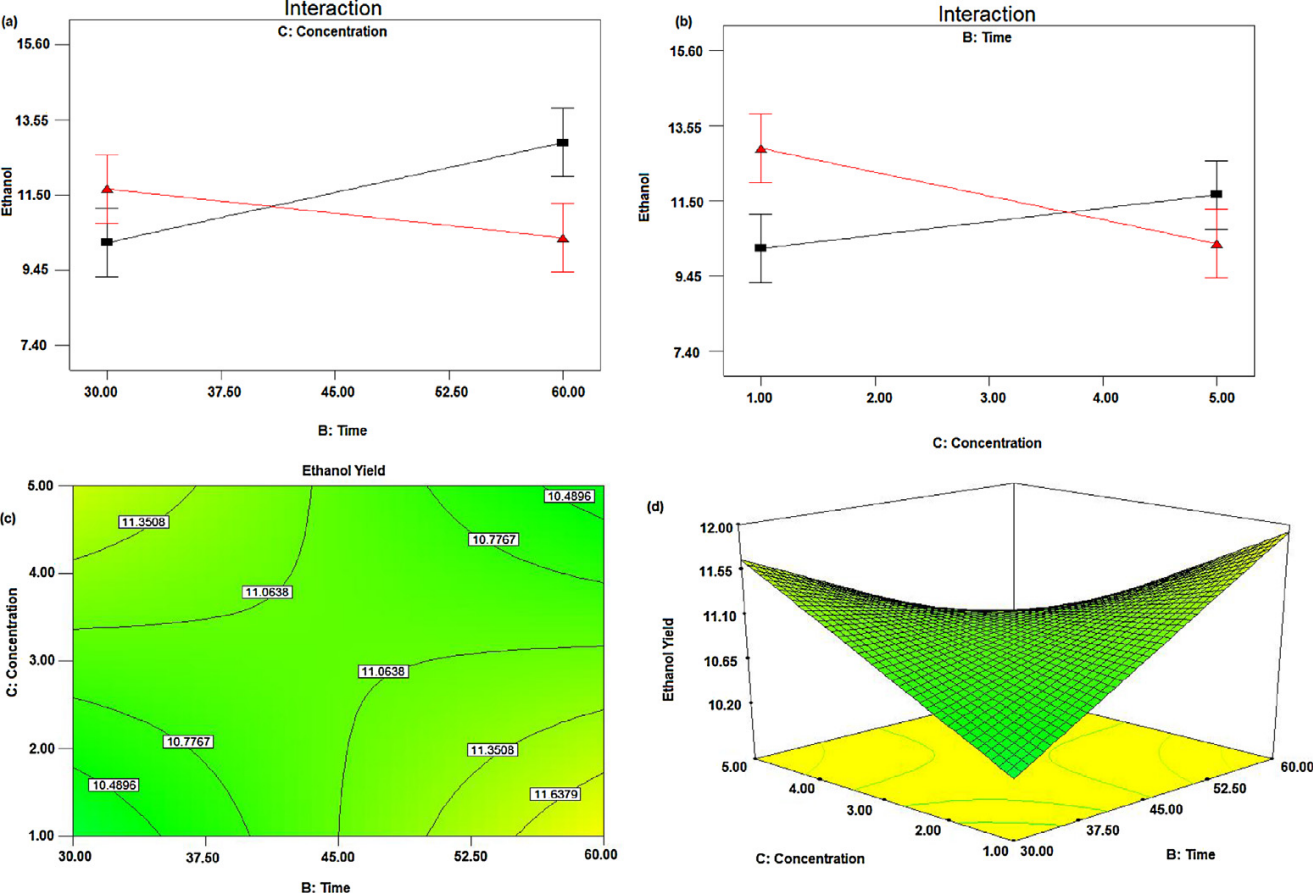
Effect of acid concentration and time on the yield of ethanol when temperature was at the center point (a) effects of time and acid concentration (fixed), (b) effects of time (fixed) and acid concentration, (c) Contour plots and (d) Response surface plots.

The effect of acid concentration and time is represented in Fig. 4(a) and (b), when hydrolysis temperature was at the center point. Ethanol yield increased with increasing acid concentration when hydrolysis time was at a low level. Similarly, ethanol yield increased with increasing hydrolysis time when the acid concentration was at a low level. This is because at low concentration and time cellulose might not be converted to fermentable sugars and at high concentration and time, the fermentable sugars might be converted to non-fermentable molecules [34]. Hence both time and acid concentration have interaction effect, in addition to the main effect for the yield of ethanol production. The contour plot graph showing predicted a response of ethanol yield as a function of hydrolysis time and acid concentration, which is shown in Fig. 4(c). Ethanol yield increased as hydrolysis time increases at lower level acid concentration and it decrease when the hydrolysis time and acid concentration became higher and higher. The response surface Fig. 4(d), obtained from hydrolysis time and acid concentration shows as hydrolysis time increases at the lower level of acid concentration and as increased level of acid concentration and lower level of time gives a positive effect on the yield of ethanol [35].

#### Effect of temperature and acid concentration

**Fig. 5:**
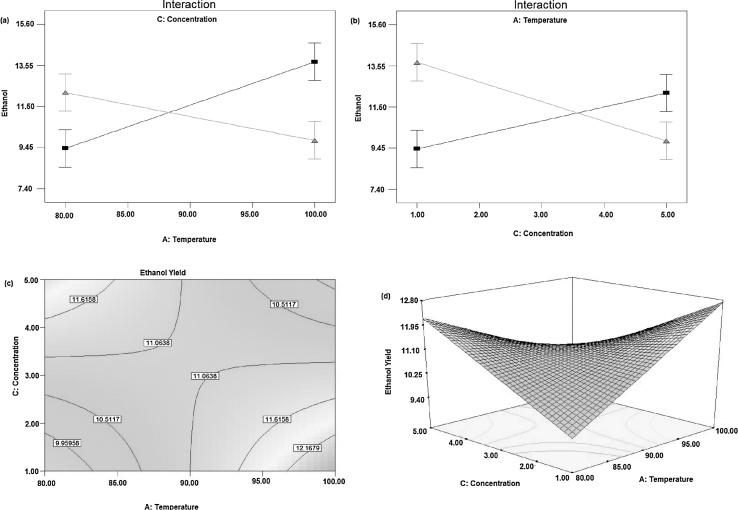
Effect of temperature and acid concentration on the yield of ethanol when time was at the center point, (a) effects of temperature and acid concentration (fixed), (b) effects of temperature (fixed) and acid concentration, (c) Contour plots and (d) Response surface plots.

The effect of temperature and acid concentration is shown in Fig. 5(a) and (b) on yield of ethanol when hydrolysis time was at the center point. Ethanol yield increased with increasing acid concentration when hydrolysis temperature was at low level and with increasing hydrolysis temperature when the acid concentration was at a low level. At lower temperature and acid concentration, the cellulose might not hydrolysis to fermentable sugars and at higher acid concentration and time the cellulose might convert to non-fermentable molecules. Hence both temperature and acid concentration have interaction effect, in addition to the main effect for the yield of ethanol production. Fig. 5(c) shows contour plot graph showing predicted response of ethanol yield as a function of hydrolysis temperature and acid concentration. The yield of ethanol increases with increasing acid concentration at the low level of hydrolysis temperature and with increasing hydrolysis temperature at a low level of acid concentration. The response surface Fig. 5(d), obtained from hydrolysis temperature and acid concentration shows ethanol yield increased with increasing acid concentration when hydrolysis temperature was at low level and with increasing hydrolysis temperature when the acid concentration was at a low level. This was consistent with the study on ethanol production from mango and banana peel reported by Taye [36]. When the above results were compiled to one, high hydrolysis time and high hydrolysis temperature would yield maximum ethanol yield at low acid concentration. This conclusion was consistent with the actual data at 1% acid concentration, 60 min hydrolysis time and 100 °C hydrolysis temperature [37]. The maximum ethanol yield found was 13.515 at (1% v, 60 min, 100 °C) of acid concentration, hydrolysis time and hydrolysis temperature respectively.

#### Optimization of hydrolysis parameters

**Fig. 6:**
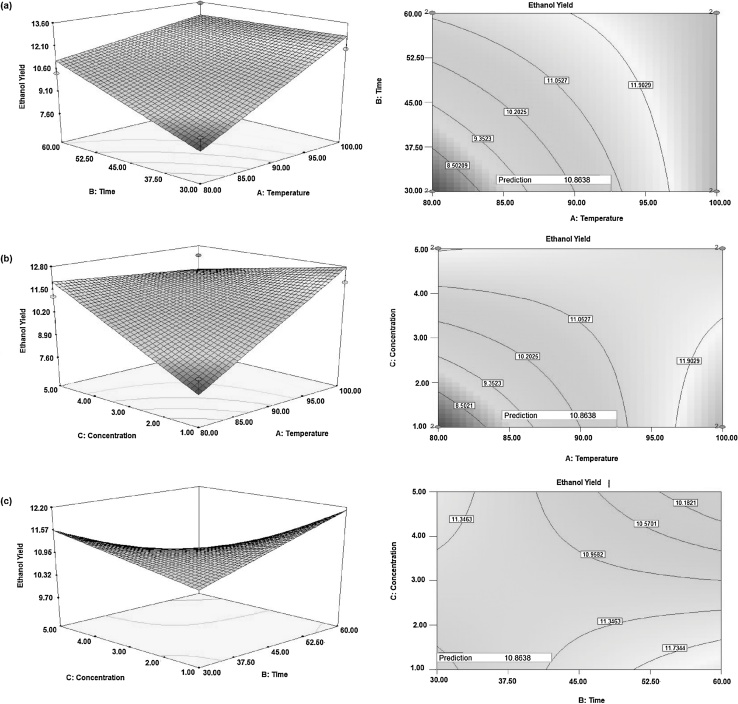
Optimization of (a) time and temperature, (b) concentration and temperature and (c) concentration and time.

The optimization of hydrolysis criteria for ethanol production from SCB using dilute acid treatment are summarized in Table 3. Design expert calculates 15 optimum possible solutions for ethanol production using different hydrolysis parameters. The optimum combinations of the three factors chosen for optimum ethanol yield (10.8538) were 92.5 °C (hydrolysis temperature), 30 min (hydrolysis time) and 1%v acid concentration. The local optimization usually requires numbers of experiments. The contours plot and response surfaces plot generated for the optimum combinations of the three factors are shown in Fig. 6(a) time and temperature, Fig. 6(b) concentration and temperature and Fig. 6(c) concentration and time. It can be seen (from Fig. 6) that consistency between the theoretical ethanol yield at the theoretical combination of parametric values and the actual result at that point, an experiment with hydrolysis acidic concentration, temperature and time were conducted at the optimized conditions. The actual result of ethanol yield at theoretical combination (10.45) was slightly lower than what was expected (10.86) [38].

#### Characterization of bagasse

**Fig. 7:**
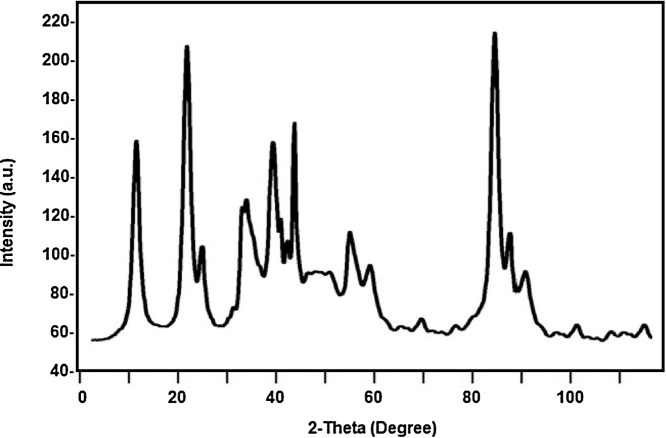
XRD of sugarcane bagasse.

The chemical analysis of sugarcane bagasse done for determination of cellulose, hemicellulose lignin ash and other composition which mention in Table 4. The X-ray diffraction study was also carried out for bagasse, shown in Fig. 7. The peak centered at 2θ = 15.5; 20.5 indicated the presence of amorphous hemicellulose and cellulose (Table 5).

**Table 4:** Optimization criteria for optimum yield of ethanol.

Parameter	Purpose	Minimum Value	Maximum value
Acid concentration (%)	Minimize	1	5
Temperature (°C)	Minimize	80	100
Time (min)	Minimize	30	60
Ethanol Yield (%)	Maximize	7.42	13.515

**Table 5:** Analysis of Sugarcane bagasse (SCB).

S.No	Components	Weight percentage
1	Total organic matter	72.6
2	Total carbonate	68.7
3	Total nitrogen	1.1
4	Cellulose	32.9
5	Hemicellulose	15.8
6	Free sugar	8.1
7	Water extractable	15.5
8	Alcohol extractable	9.4
9	lignin	28.4
10	Ash	3.2

## Outcomes

The outcome of this research methodology is that sugarcane bagasse is promising lignocellulosic feedstock for bioethanol production. One of the most important factors in the acid treatment of lignocellulose is the determination of optimal conditions required to provide the maximum yield of fermentable sugars and the least amount of inhibitors. All the three hydrolysis parameters were significant variables for the yield of ethanol. The yield of ethanol decreases at very high and low hydrolysis temperature, hydrolysis time and acid concentration. The study showed that response surface methodology is the reliable tool for optimizing the pretreatment of biomass for ethanol production. Maximum 10.86 ml/50 g of ethanol were yield at optimum 92.59 °C hydrolysis temperature, 30 min time, 1% acid concentration. The quality of the fit polynomial model was expressed by the coefficient of determination R^2^ (Adj-R^2^ = 0.7252 and Pre-R^2^ = 0.5362) and its statistical significance was checked by the Fisher *F*-test. Model terms were selected or rejected based on the P-value (probability) with 95% confidence level. Three-dimensional plots and their respective contour plots were obtained based on the effects of the levels of three factors. Finally, ethanol production from sugarcane bagasse is doubtlessly an attractive business from the economic and environmental point of view.

## Contribution

Both authors have equal contribution for experiment work.
